# The rhythm of retinoids in the brain

**DOI:** 10.1111/jnc.12620

**Published:** 2013-12-15

**Authors:** Jemma Ransom, Peter J Morgan, Peter J McCaffery, Patrick N Stoney

**Affiliations:** * Institute of Medical Sciences, School of Medical Sciences, University of AberdeenAberdeen, UK; † Rowett Institute of Nutrition and Health, University of AberdeenAberdeen, UK

**Keywords:** circadian, neural plasticity, nuclear receptor, photoperiod, retinoic acid, vitamin A

## Abstract

The retinoids are a family of compounds that in nature are derived from vitamin A or pro-vitamin A carotenoids. An essential part of the diet for mammals, vitamin A has long been known to be essential for many organ systems in the adult. More recently, however, they have been shown to be necessary for function of the brain and new discoveries point to a central role in processes ranging from neuroplasticity to neurogenesis. Acting in several regions of the central nervous system including the eye, hippocampus and hypothalamus, one common factor in its action is control of biological rhythms. This review summarizes the role of vitamin A in the brain; its action through the metabolite retinoic acid via specific nuclear receptors, and the regulation of its concentration through controlled synthesis and catabolism. The action of retinoic acid to regulate several rhythms in the brain and body, from circadian to seasonal, is then discussed to finish with the importance of retinoic acid in the regular pattern of sleep.

We review the role of vitamin A and retinoic acid (RA) as mediators of rhythm in the brain. In the suprachiasmatic nucleus and hippocampus they control expression of circadian clock genes while in the cortex retinoic acid is required for delta oscillations of sleep. Retinoic acid is also central to a second rhythm that keeps pace with the seasons, regulating function in the hypothalamus and pineal gland.

Vitamin A is a vital component of the mammalian diet that is delivered to tissues in the form of circulating retinol (Fig.1, structure 2). Retinol is metabolized to the transcriptionally active compound retinoic acid (RA; Fig.1, structure 5). That RA is a key molecule during embryonic development is well established (Ross *et al.* 2000) and it is particularly crucial during development of the central nervous system (CNS; Maden 2002). More recently, however, results point to an important role for RA in the adult CNS and evidence suggests that RA mediates neuronal plasticity (McCaffery *et al.* 2006; Chen *et al.* 2012) as well as neurogenesis in both the hippocampus (Goodman *et al.* 2012) and hypothalamus (Shearer *et al.* 2012b). Now, a new homeostatic role is emerging for RA as a regulator of biological rhythms within the CNS (Fig.2).

**Fig 1:**
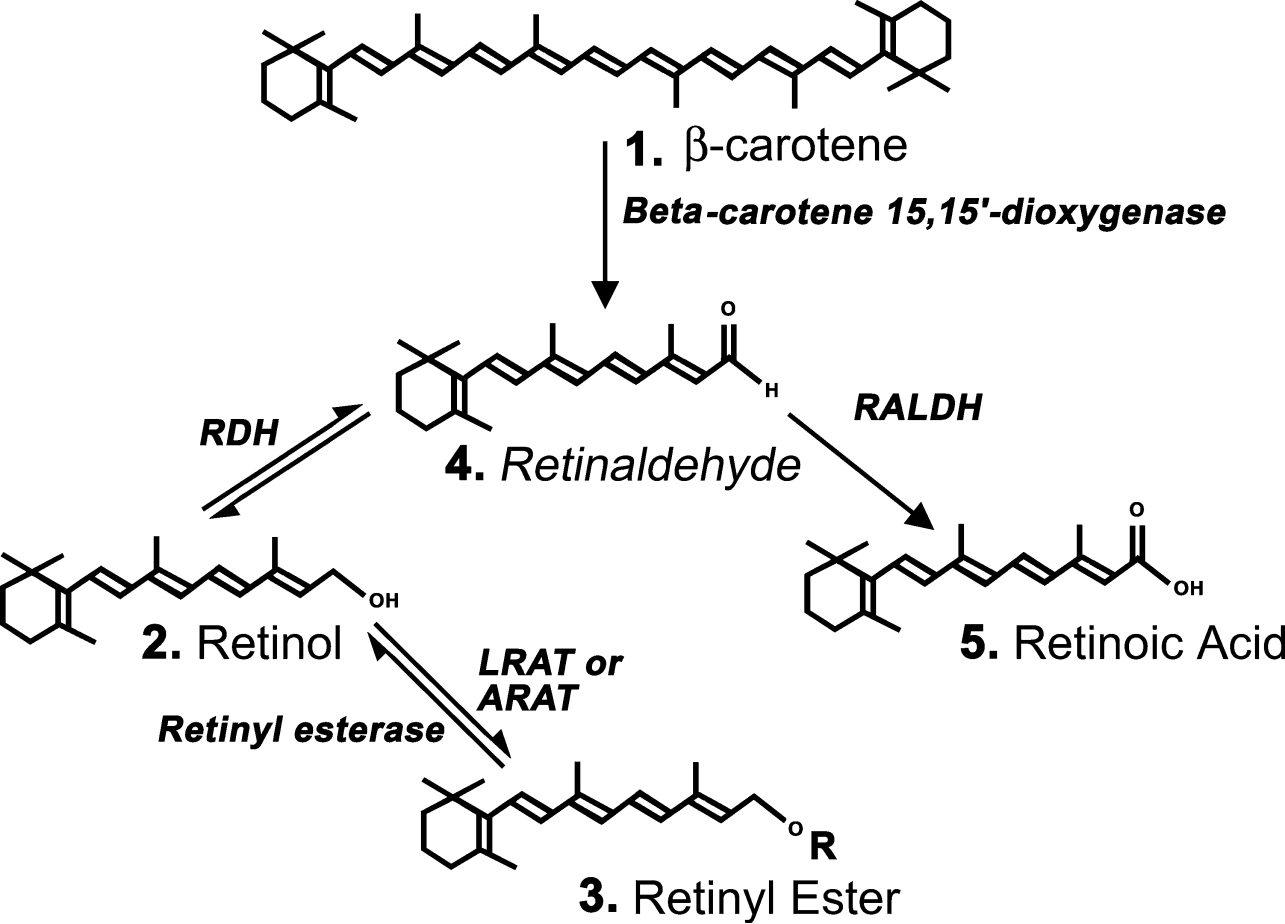
Chemical structures of retinoid family members. β-carotene (structure 1) is cleaved by β-carotene 15,15′-monooxygenase to form two molecules of retinaldehyde (structure 4). The double arrow between retinol (structure 2) and retinaldehyde (structure 4) indicates the interconversion between the two retinoids catalyzed by retinol dehydrogenases (RDHs) primarily RDH1 and RDH10 and dehydrogenase/reductase (SDR family) member 9 (DHRS9). The single arrow between retinaldehyde (structure 4) and retinoic acid (structure 5) indicates the irreversible oxidation catalyzed by retinaldhyde dehydrogenase (RALDH) family members. Esterification of retinol is carried out by lecithin:retinol acyltransferase (LRAT) or acyl-CoA:retinol acyltransferase (ARAT). The predominant retinyl ester (structure 3) is retinyl palmitate (*r* = C_15_H_31_).

**Fig 2:**
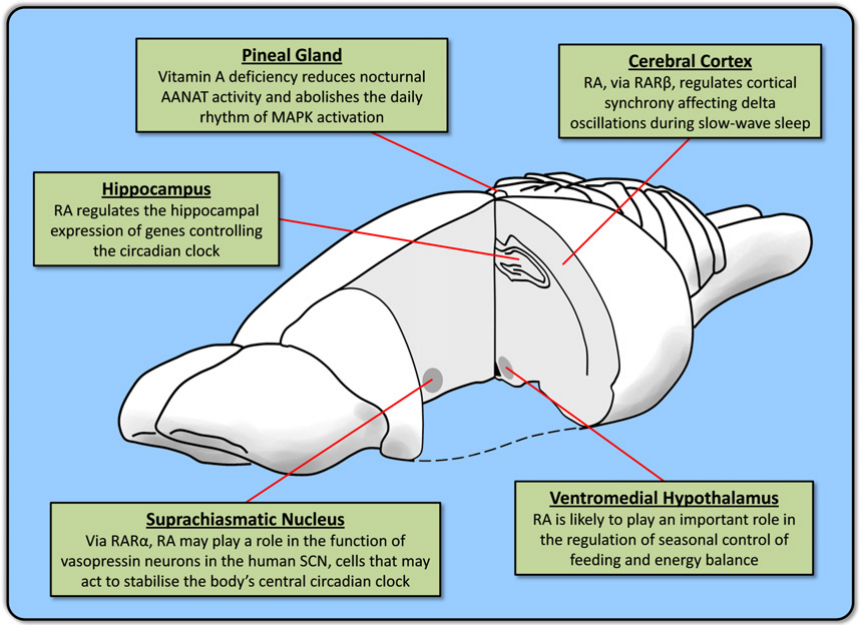
Retinoic acid in the CNS regulates biological rhythms. Retinoic acid (RA) may contribute to the regulation of circadian rhythms in the suprachiasmatic nucleus (SCN), the central clock of the body (Meng *et al.* 2011), the pineal gland (Herbert and Reiter 1985; Guillaumond *et al.* 2005), which signals day/night to the rest of the body, and peripheral clocks such as that of the hippocampus (Golini *et al.* 2012). There is also considerable evidence suggesting that RA plays a crucial role in the seasonal rhythms of energy balance in the ventromedial hypothalamus (Anzano *et al.* 1979; Ross *et al.* 2005; Shearer *et al.* 2010). Rhythmic EEG oscillations in the cerebral cortex during sleep are altered by RA signalling (Maret *et al.* 2005).

It is now evident that several components of the RA signalling pathway oscillate according to photoperiodic changes in light conditions. Increased day length leads to enhanced retinoid signalling within the hypothalamus of the F344 photoperiodic rat (Shearer *et al.* 2010). The daily peak in melatonin synthesis by the pineal gland is known to be reduced under vitamin A-deficient conditions in the Japanese quail (Fu *et al.* 1999). In addition, polymorphisms in the RA receptor β gene can regulate the relative contribution of delta oscillations to slow-wave sleep (Maret *et al.* 2005). These observations point to roles for RA in both circadian and seasonal rhythms, and also in the cortical regulation of sleep. This review will discuss the mechanisms that link RA with these biological rhythms within the CNS.

## The vitamin A signalling pathway

Vitamin A and related retinoid compounds (Fig.1) have a similar chemical structure containing a 6 carbon β-ionone ring and an isoprenoid chain termed a retinyl group. Mammals are not able to generate vitamin A *de novo* and must instead consume plant or animal sources of this dietary nutrient. Vitamin A is vulnerable to loss through oxidation and in animal tissues is found in the more stable form of fat-soluble retinyl esters. Vitamin A can also be obtained from plant food sources such as β-carotene, which can be cleaved by the enzyme β-carotene 15,15′-monooxygenase to retinaldehyde (Fig.1).

Retinol derived from either dietary source is absorbed by cells of the intestinal mucosa where it is bound by cellular retinol binding protein II (CRBP-II; Noy 2000). CRBP-II then specifically directs retinol substrate to the enzyme lecithin:retinol acyltransferase (Fig.1; Herr and Ong 1992), resulting in the re-esterification of retinol to retinyl esters. These are packaged with other dietary lipids into chylomicrons and secreted from the intestinal mucosa into the general circulation (Blomhoff *et al.* 1990). The liver is the principal site for the storage and metabolism of retinoids. Here, hydrolysis of retinyl esters results in the formation of retinol which associates with retinol-binding protein (RBP; Newcomer and Ong 2000). Binding of retinol to RBP results in secretion of retinol-RBP into the blood plasma for transportation to peripheral tissues (Ronne *et al.* 1983).

### Retinoic acid synthesis

The transport of retinol in the general circulation coupled with its lipophilic nature renders retinol available to virtually all cells. However, not all tissues can oxidize retinol to RA; only those with a requirement for RA will do this. The ability of cells to synthesize RA and therefore to initiate retinoid signalling is determined by the expression of RA synthetic enzymes. Fig.1 illustrates cellular RA synthesis in two oxidative stages (Napoli 2012). Retinol is first converted to retinaldehyde, a reversible reaction catalysed by retinol dehydrogenases (RDHs) primarily RDH1 and RDH10 and dehydrogenase/reductase (SDR family) member 9 (DHRS9). Retinaldehyde is then converted to RA in an irreversible reaction catalysed by retinaldehyde dehydrogenases (RALDH1, 2 and 3, also called ALDH1A1, 2 and 3; McCaffery *et al.* 1992; Suzuki *et al.* 2000).

In both mouse and human, the three isoforms of RALDH are highly expressed by tissues of the developing embryo. RALDH1 is specifically expressed in the dorsal retina where RA signalling contributes to dorsoventral patterning (McCaffery *et al.* 1999), and is also present in the axons and terminals of a subset of tyrosine hydroxylase-expressing dopaminergic neurons of the mesostriatal and mesolimbic system. These neurons form a RA-generating projection from the substantia nigra to the corpus striatum and nucleus accumbens (McCaffery and Drager 1994; Jacobs *et al.* 2007). RALDH2 is widely expressed throughout the embryo during development; *RALDH2*^*-/-*^ embryos fail to develop beyond E8.5, demonstrating the essential role of RALDH2 in development (Niederreither *et al.* 1999). RALDH3 expression is confined to the ventral retina (McCaffery *et al.* 1992) and the lateral ganglionic eminence of the telencephalon (Li *et al.* 2000). Here, RA signalling regulates cell proliferation, migration and fate determination (Molotkova *et al.* 2007; Crandall *et al.* 2011).

The RALDHs are key to the function of RA in the embryonic CNS and are strongly expressed in a variety of locations during embryogenesis (McCaffery *et al.* 1992, 1999; McCaffery and Drager 1994). However, in the adult rodent, there is a stark reduction in the number of tissues that express RALDHs, and by implication the number of tissues able to generate RA and participate in RA signalling. These limited regions include the hypothalamus in which the RALDHs are expressed in tanycytes to potentially control hypothalamic regulation of energy balance (Shearer *et al.* 2010) by mechanisms that include regulation of neurogenesis (Shearer *et al.* 2012b). In the hippocampus, RA is synthesized from retinaldehyde by the high levels of RALDH2 expressed in the meninges immediately adjacent to the dentate gyrus, which RA is presumed to enter by diffusion (Goodman *et al.* 2012). This is possible in the rodent hippocampus because of its relatively small size, but would be ineffective in supplying the much larger human brain. In the human hippocampus, high levels of all three RALDH enzymes are expressed by the neurons themselves in both the dentate gyrus and cornus ammonis (Fragoso *et al.* 2012). This potentially provides high levels of RA in the human hippocampus and the dynamics of RA supply in the human brain are likely different from that in the rodent. The discovery of widespread RA synthesis by neurons in the human hippocampus serves to emphasize that RA is crucial for hippocampal function in all mammalian species, regulating the neuroplasticity essential for learning and memory (Shearer *et al.* 2012a).

### The actions of retinoic acid – regulation of transcription

Upon synthesis, the primary action of RA is to regulate the transcription of target genes, which is achieved through the activation of nuclear receptors. The retinoic acid receptors (RARα, β and γ) are ligand-induced transcription factors activated by the all-*trans* isomer of RA. Retinoic acid binding to RAR results in conformational changes within the ligand binding domain (LBD), promoting interaction with a number of co-regulator complexes to predominantly activate, but sometimes repress, transcription the latter also occurring in the absence of ligand (Rochette-Egly and Germain 2009). The RAR heterodimerizes with the retinoid X receptor (RXRα, β and γ). Although RXR can be bound by the 9-*cis* isomer of RA (Chambon 1996), this isomer is present in a very limited number of organs (Kane 2012) and given that ligand-bound RAR can effectively promote transcription with an RXR partner lacking bound ligand, it may commonly function in that way.

### Retinoic acid regulation of transcription – new retinoic acid response elements

The RAR/RXR heterodimers regulate transcription by binding to DNA elements consisting of two direct repeats of an RGKTCA motif (R = A/G, K = G/T) traditionally viewed to have a spacing of 1, 2 or 5 bp (DR1, DR2 and DR5; reviewed by Bastien and Rochette-Egly 2004). In recent years, however, techniques such as ChIP-chip and ChIP-seq have allowed the large-scale study of DNA sequences bound by RARs. These studies suggest that the earlier described consensus is broadly correct, but also demonstrate the remarkable flexibility of RARs in binding a wide variety of sequences and suggest that the sites bound by RARs are highly cell type-specific. DR5 was identified as the most common RARE in MCF-7 cells (Hua *et al.* 2009) with a very low frequency of RAR-bound DR2 elements, while DR2 and DR5 were the most RAR-occupied sites in embryonic stem (ES) cells during early RA-induced differentiation (Mahony *et al.* 2011). However, other similar studies found that many sites occupied by RARs do not fit the canonical DR1, DR2 or DR5 consensus (Delacroix *et al.* 2010; Moutier *et al.* 2012). In another study using RA-treated mouse ES cells and F9 cells, DR0 elements were found to be the most common site bound by RARs (Moutier *et al.* 2012). DR8 was also found to be a common site of RAR binding, occurring with a similar frequency to DR5. Around 50% of DR8 RAREs incorporated a third motif within the other two, forming a compound RARE consisting of overlapping DR2 and DR0 sites. Analysis of these compound RAREs demonstrated that RAR-RXR heterodimers are able to bind the DR0, DR2 and DR8 elements and that DR8 sequences can act as RA-responsive elements, but also suggested that the DR2 and DR0 sites of the compound RAREs are preferentially occupied relative to the DR8. Although preferentially bound, a DR0-type RARE did not confer RA-responsiveness in a reporter assay. Therefore, despite being the most common site of RAR binding, DR0 RAREs do not appear to act as independent transcriptional regulators.

Prior to this analysis, several DR0-type RAREs had been described. In the context of the brain, one such DR0 RARE has been identified in the promoter of *glial fibrillary acidic protein* (*GFAP*), approximately 2.5 kb upstream of the transcription start site (Asano *et al.* 2009). RA acted synergistically with leukaemia inhibitory factor (LIF) to induce *GFAP* expression during differentiation of neural precursor cells to astrocytes, but the effect of RA in the absence of LIF was small, supporting the observation of Moutier *et al*. (2012) that DR0 RAREs do not act independently. RA increased histone acetylation in the *GFAP* promoter, facilitating the LIF-induced binding of pSTAT3, and the synergistic effect of RA on LIF could be mimicked using the histone deacetylase inhibitor valproic acid. Other potential DR0-type RAREs have been identified in the promoters of the rat genes encoding oxytocin (Adan *et al.* 1993) and vasopressin (*AVP*; Burbach *et al.* 1993). RAR-occupied DR0/8 elements, but not DR5s, were frequently located along with other conserved motifs within a larger regulatory element (Moutier *et al.* 2012). Therefore, RARs may act via DR0-type RAREs as enhancers of the action of other transcriptional regulators, perhaps through epigenetic modifications of their binding sites.

### Additional actions of retinoic acid – regulation of protein translation and kinase activity

In addition to the canonical role of RARs as transcription factors, it is becoming increasingly apparent that RARs, in particular RARα, can have non-genomic functions. These mechanisms include regulation of protein translation. Cytoplasmic RARα regulates the translation of synaptic proteins, including the α-amino-3-hydroxy-5-methyl-4-isoxazolepropionic acid (AMPA) receptor subunit glutamate receptor 1 (GLUR1) and RARα appears to directly bind to sequences in the 5′ UTR of the *GLUR1* mRNA (Poon and Chen 2008; Chen *et al.* 2008; reviewed in Chen *et al.* 2012). RARα interacts with mRNAs via its LBD and the binding of RA disrupts the interaction and relieves repression of translation. Most recently, evidence has been published showing that, through RARα, RA may also non-genomically regulate inhibitory synaptic activity via the control of membrane trafficking of GABA receptors (Sarti *et al.* 2013).

It is evident from such control of translation that RARs can function in the cytoplasm, in addition to their regulatory role to control transcription in the nucleus. A series of reports have described an additional extranuclear function for RARs in the control of kinase activity that can lead to rapid cellular changes, often within minutes. During neuronal differentiation, RARγ can act in the cytoplasm to regulate neurite outgrowth via a direct interaction with c-SRC (Dey *et al.* 2007). The RARs can also function in the plasma membrane. RARα directly interacts with phosphatidylinositol-3-kinase in the plasma membrane to activate extracellular signal-regulated kinase 1/2 (ERK1/2) MAPK signalling pathways (Masiá *et al*. 2007). Alternatively RARα can associate with G protein αq within lipid rafts to activate the p38MAPK/MSK1 pathway (Piskunov and Rochette-Egly 2012). ERK1/2 MAPK pathways are activated in neuronal cells and embryonic stem cells by RA (Bost *et al.* 2002; Masiá *et al*. 2007), while in other cells types p38MAPK is activated by RA (Alsayed *et al.* 2001; Piskunov and Rochette-Egly 2012). This latter pathway can be upstream of activation of MSK1 (Bruck *et al.* 2009) and provides one mechanism by which a rapid action by RA to promote protein phosphorylation then feeds back to RARs more traditional role of control of transcription. These non-genomic and genomic mechanisms intersect as a result of MSK1′s phosphorylation of RARα, promoting its capacity to induce gene transcription. Overall, these non-genomic pathways likely provide an important route by which RA has rapid control of cellular events such as neurite outgrowth (Dey *et al.* 2007), growth cone turning (Farrar *et al.* 2009) and control of neuronal differentiation (Cañón *et al*. 2004).

### Retinoic acid catabolism

The concentration of RA available to activate the RAR/RXR complex and therefore to contribute to retinoid signalling within tissues is determined by the relative expression of both the synthetic and degradative enzymes. Endogenous degradation of RA is primarily via oxidation to a number of polar metabolites including 4-oxo-RA and 4-hydroxy-RA. A number of cytochrome P450 enzymes are able to catalyse this reaction, including CYP2C8, CYP3A4 and CYP2C9 (Marill *et al.* 2000). However, the high *K*_M_ values of these reactions mean that their relative contributions to RA metabolism *in vivo* are likely to be negligible (Gomaa *et al.* 2011).

The principal CYP450 family responsible for RA degradation *in vivo* is CYP26. There are three known isoforms encoded by separate genes: CYP26A1, CYP26B1 and CYP26C1. The CYP26s sequentially oxidize RA to more polar metabolites such as 4-hydroxy-RA and 4-oxo-RA using oxygen and nicotinamide adenine dinucleotide phosphate reduced (NADPH) co-factors (McCaffery and Simons 2007; Topletz *et al.* 2012). In the embryo, the CYP26 enzymes play a role in determining the cellular exposure to RA by inactivating it in cells that do not participate in retinoid signalling, or where RA-activated gene transcription would be detrimental to the cell, for example through the perturbation of the developmental programme (Thatcher and Isoherranen 2009). All three enzymes are expressed during mammalian development in a highly regulated spatio-temporal pattern (Kudoh *et al.* 2002). During development, CYP26A1 and CYP26B1 are essential for generating an uneven distribution of RA necessary for the development of the hindbrain in the mouse embryo (Abu-Abed *et al.* 2001). Furthermore, CYP26A1 expression in the prospective head region of the gastrulating mouse embryo is vital for the formation of anterior structures, as RA receptors in this region must be in an unliganded state to allow the proper development of the head (Ribes *et al.* 2006).

In the adult, CYP26A1 expression is much sparser with only low expression found in the brain (Wang *et al.* 2002). In contrast, CYP26B1 is considered a brain-specific RA-degrading enzyme with a high level of expression found in the adult cerebellum (Trofimova-Griffin and Juchau 2002) and hippocampus (Abu-Abed *et al.* 2002). *In situ* hybridization analysis found at the Allen Brain Atlas database shows an even more widespread distribution including the cortex and amygdala (http://www.brain-map.org/).

Taken together, net RA production is determined by the relative expression of RA synthetic and degradative enzymes in a given tissue. A further element of control is demonstrated by the ability of RA to induce the CYP26 enzymes and therefore limit its own production via a negative feedback pathway (Reijntjes *et al.* 2005). *CYP26A1* is regulated directly by RA, its promoter containing a functional RARE (Loudig *et al.* 2000, 2005; Hu *et al.* 2008). This is a key regulatory mechanism necessary to achieve appropriate vitamin A homeostasis.

## Retinoic acid in the regulation of circadian and seasonal rhythms

Animals are able to anticipate change in the environment over both the 24-h day and through the changing seasons by the use of a variety of rhythmic systems. The 24-h circadian clock is based on a central rhythm generator in the suprachiasmatic nucleus (SCN), acting as a master clock for the body, although most tissues also contain their own peripheral clock (Dibner *et al.* 2010). These clocks rely on an internal oscillation network within the cell reliant on a set of feedback loops (Fig.4), but entrained by daylight to keep the rhythm synchronized with environmental day and night. For seasonal rhythms the synchronizing cue is photoperiod, the annual cycle of changing day length. Photoperiodic information is then conveyed to the whole organism through the secretion of melatonin from the pineal gland. Secretion of melatonin at night is a continuous signal, the duration of which reflects the length of the subjective night (Morgan and Hazlerigg 2008).

Both systems are dependent on light: the circadian system is entrained by daylight while photoperiodic information relies on day length. Of course the eye is essential for both of these and, in turn, vitamin A is the key to both eye development and visual function. RA patterns retinal development (McCaffery *et al.* 1999) while in the photoreceptors retinaldehyde is the chromophore for visual pigment essential for visual transduction via photoreceptors, but is also the chromophore for melanopsin in the intrinsically photosensitive retinal ganglion cells that innervate and signal to the SCN (Palczewski 2012). The evolution of retinoid function as part of this system may have overlapped with the involvement of retinoids in other rhythmic systems in the body and brain. The roles of RA in both circadian and seasonal rhythms are outlined below.

### Circadian rhythms in the suprachiasmatic nucleus and hippocampus

**Fig 3:**
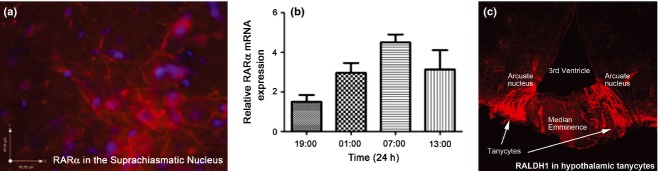
Rhythmic retinoic acid signalling proteins in the hypothalamus. (a) Immunohistochemistry shows that retinoic acid receptor (RAR)α protein is present in both cell bodies and processes in the adult rat suprachiasmatic nucleus. (b) mRNA expression of the encoding gene, *RARA*, appears to oscillate through the 24-h day in the rat suprachiasmatic nucleus. (c) Retinaldehyde dehydrogenase (RALDH)1, the retinoic acid (RA)-synthesizing enzyme which shows seasonal changes in rat hypothalamic tanycytes, is also strongly expressed in ventral tanycytes in the mouse hypothalamus.

**Fig 4:**
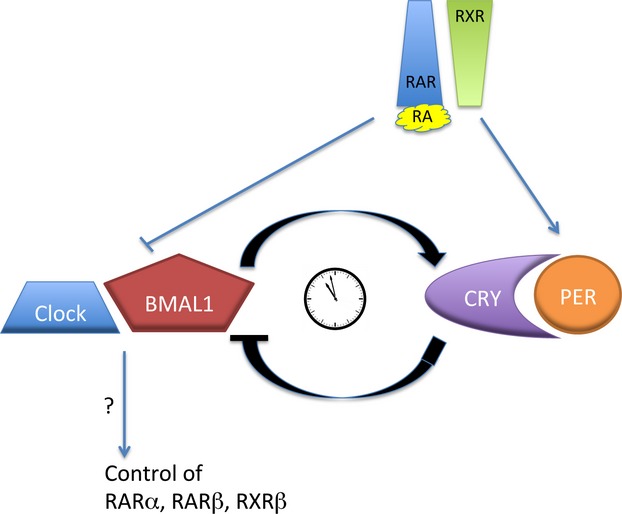
The influence of retinoic acid (RA) on the circadian oscillator. The core set of proteins that create the cellular circadian pacemaker achieve this by forming a positive and a negative feedback loop (Ko and Takahashi 2006). The positive loop involves a heterodimer of CLOCK:BMAL1. These activate transcription of target genes that contain an enhancer box (E-box) sequence, such as PER and CRY. The opposing half of the circuit is the PER and CRY heterodimer which exerts a repressive action on CLOCK:BMAL transcriptional activity. In addition, not shown in the diagram, are multiple interlocked feedback loops that likely both stabilize and provide robustness to the rhythm. These additional loops include pathways that require retinoic acid receptor-related orphan receptors (RORs). RA is reported to influence the central components of the circadian feedback loop by inhibiting the expression or activity of CLOCK/BMAL (McNamara *et al.* 2001; Shirai *et al.* 2006) and inducing expression of PER (Shirai *et al.* 2006). In turn, the genes that encode retinoic acid receptors (RAR)α, β and retinoid X receptor (RXR)β may be regulated by CLOCK/BMAL via their E-box sites (Navigatore-Fonzo *et al.* 2013).

RA may influence the body's central clock as high levels of RARα expression are found in the vasopressin-expressing neurons of the human SCN (Meng *et al.* 2011) and our own preliminary studies show RARα present in processes and cell bodies in the SCN while mRNA transcript levels cycle with a circadian rhythmicity (Fig.3a, b). The function of vasopressin neurons in the SCN was not clear until a recent study demonstrated that mice lacking the V1a/V1b vasopressin receptors are able to adjust rapidly to phase-shifts in the light-dark cycle, making them extremely resistant to jet lag (Yamaguchi *et al.* 2013) and this was replicated by pharmacological blockade of V1a/b. This indicates that vasopressin signalling in the SCN may stabilize the central circadian clock. In a screen of nuclear receptor regulators of circadian genes RA was found to induce expression of *AVP* (the vasopressin gene), as well as the clock genes *PER1* and *PER2*, via an enhancer box (E-box)-dependent mechanism (Shirai *et al.* 2006). PER proteins are central to the cellular circadian rhythm through reciprocal positive and negative feedback loops acting via dimers of CLOCK:BMAL1 and CRY:PER (Fig.4). The results of Shirai *et al*. also suggested that RA could inhibit expression of CLOCK:BMAL1, although only when RARα was present. This supports an earlier study on blood vessels in which RA could inhibit CLOCK:BMAL1 dimer transcriptional activity (as well as that of MOP4:BMAL1), acting via a RARα:RXRα heterodimer (McNamara *et al.* 2001). Thus, from these studies, RA may promote CRY:PER and inhibit CLOCK:BMAL1 activity (Fig.4).

RA may also play a role in the regulation of circadian genes in the hippocampus, given that vitamin A deficiency can phase shift the expression patterns of the clock gene *BMAL1* and completely abolishes the circadian expression of *PER1* (Golini *et al.* 2012; Navigatore-Fonzo *et al.* 2013). Phase-shifting effects of RA are also evident in the capacity of RA to alter rhythmic expression of vascular *PER2* both *in vitro* and *in vivo* (McNamara *et al.* 2001). Further implying the involvement of RA in circadian control in the hippocampus is the finding that many of the components of the retinoid signalling pathway show a significant diurnal oscillation (Navigatore-Fonzo *et al.* 2013). Of the potential downstream elements that RA may control in the hippocampus, the circadian rhythm of *brain-derived neurotrophic factor (BDNF)* is disrupted in response to vitamin A deficiency (Golini *et al.* 2012).

The E-box is one of the key enhancers used to control gene expression of several of the circadian genes and is the target of CLOCK:BMAL1. Of the genes required to transduce the RA signal, perfect E-box or E-box-like sites are found in *RARA, RARB* and *RXRB* making these genes potential outputs of the circadian cycle (Navigatore-Fonzo *et al.* 2013). Conversely, RAREs were found in the promoter regions of *BMAL1* and *PER1* (Golini *et al.* 2012), suggesting that there may be direct transcriptional regulation of these genes by RA (Navigatore-Fonzo *et al.* 2013).

### Circadian cycles controlled by the pineal gland

The pineal gland receives information about day and night from the retina via the suprachiasmatic nucleus (SCN), the central clock that synchronizes the functions of the body. There is some evidence favouring a role for RA signalling in the generation of circadian rhythms by the mammalian body clock. Nocturnal activity of arylalkylamine *N*-acetyltransferase, the rate-limiting enzyme in the synthesis of melatonin from serotonin, is greatly reduced in the pineal gland of vitamin A-deficient rats (Herbert and Reiter 1985), although the circadian timing of the nocturnal increase in melatonin appeared unaffected. Expression of *RORB*, which encodes a retinoid orphan receptor that binds RA with high affinity, is increased at night in the pineal gland (Bailey *et al.* 2009); genes encoding the retinoid-binding proteins RBP3 and transthyretin (TTR) are also expressed. RA is known to regulate the MAP kinases and the circadian activation of ERK1/2 is lost in the pineal of vitamin A-deficient (VAD) rats (Guillaumond *et al.* 2005). This function of vitamin A was found to be SCN-dependent, but independent of the superior cervical ganglion (SCG) neurons, raising the possibility that blood-borne nutrients are able to alter the function of the pineal gland.

### Seasonal rhythms

Certain species display marked physiological responses to seasonal change in body weight, food intake and reproductive status. These changes are driven by the hypothalamus and the duration of nocturnal melatonin synthesis is the means by which the mammalian hypothalamus determines the season. When the night is short, the reduction in melatonin disinhibits the release of thyroid-stimulating hormone from the *pars tuberalis* of the pituitary (Ono *et al.* 2008), up-regulating thyroid hormone signalling in the hypothalamus. Hypothalamic thyroid hormone signalling downstream of melatonin appears to be crucial for seasonal responses in birds (Yoshimura *et al.* 2003) and mammals (Barrett *et al.* 2007; Ross *et al.* 2009).

There is evidence that vitamin A and RA are mediators of seasonal changes in feeding and energy balance though their actions in the hypothalamus. Evidence for this comes from earlier studies showing that vitamin A deficiency in rats causes a decrease in food intake accompanied by a rapid drop in bodyweight which persists even after force-feeding (Anzano *et al.* 1979). Also, mice lacking the RA synthesizing enzyme RALDH1 are extremely resistant to diet-induced obesity (Ziouzenkova *et al.* 2007). These two observations suggest a pivotal role for RA in the control of energy balance and more recently, the hypothalamus, the region of the brain controlling growth and feeding, has been recognized as a site of RA action (Ross *et al.* 2004; Shearer *et al.* 2010, 2012b; Helfer *et al.* 2012).

The RA synthetic enzymes RALDH1 and RALDH2 are found in rat tanycytes (RALDH1 only in mice; Shearer *et al.* 2010 and Fig.3c), cells which line the third ventricle adjacent to the hypothalamus and are thought to be important for the transduction of photoperiodic signals into the hypothalamic nuclei. The tanycytes project RALDH1-containing processes into the arcuate nucleus and the dorsomedial hypothalamic nucleus (Shearer *et al.* 2012b). RA synthesis by RALDH1/2 in the tanycytes is sensitive to photoperiod with significantly higher levels of RA in the hypothalamus of F344 rats maintained on summer-like long day photoperiods relative to short days (winter; Helfer *et al.* 2012). Such a seasonal change in RA production has recently been summarized by Shearer *et al*. (Shearer *et al.* 2012a). This is in contrast to hypothalamic T3, which shows little change with photoperiod (Ross *et al.* 2011).

A further indicator of the photoperiodic role of RA in the hypothalamus has been demonstrated in Siberian hamsters and F344 rats in which a long day photoperiod results in large up-regulation of many genes involved in retinoid signalling, including those encoding extracellular and intracellular retinoid-binding proteins (*STRA6*, *TTR*, *CRABP1*, *CRABP2*, *CRBP1*), retinoic acid receptors (*RAR*) and RA-degrading enzymes (*CYP26B1*; Ross *et al.* 2004, 2005; Shearer *et al.* 2010; Helfer *et al.* 2012). Intriguingly, *RPE65*, previously thought to be restricted to the retinal pigment epithelium of the eye, is also expressed and photoperiodically regulated in the cells lining the third ventricle in the rat (Helfer *et al.* 2012). In the retinal pigment epithelium, RPE65 catalyses the isomerization of all-*trans* retinol to 11-*cis* retinaldehyde and its expression may be a vestige of photoreceptor-like cells in the mammalian hypothalamus. Deep-brain photoreceptors are present in many non-mammalian species (Vigh *et al.* 2002) and the periventricular region of the hypothalamus of birds is known to detect light directly (Thayananuphat *et al.* 2007). These changes suggest a large seasonal up-regulation of RA signalling in the hypothalamus during periods of increased somatic growth and feeding. Many of these changes in gene expression precede physiological changes and, together with the effects of vitamin A deficiency on energy balance (Anzano *et al.* 1979), this implies a causative role for RA (Ross *et al.* 2005).

The targets of RA in the hypothalamus are currently not well understood. Some potential targets of RA have been previously discussed (Shearer *et al.* 2012a). Recently, it has been found that components of the Wnt signalling pathway are photoperiodically regulated in the hypothalamus of F344 rats, with expression of two inhibitors of Wnt signalling, *DKK3* and *SFRP2*, increased in long day photoperiods when hypothalamic RA levels are high (Helfer *et al.* 2012). Wnt signalling and RA signalling are both involved in adult neurogenesis in the hippocampus (Maden 2007; Inestrosa and Arenas 2010), and there is some evidence that Wnt signalling components are regulated by RA during neurogenesis and neuronal differentiation (Katoh 2002; Jacobs *et al.* 2006). In F9 teratocarcinoma cells, *SFRP2* is specifically up-regulated by RARγ in response to RA (Su and Gudas 2008). In the medial arcuate nucleus, the number of cells expressing nuclear RARγ, suggestive of an increase in transcriptional activity, was increased in rats in long day photoperiods compared to short day (Shearer *et al.* 2010). Such evidence hints at the Wnt signalling pathway as one mediator of RA's action in the hypothalamus (Helfer *et al.* 2012).

### Vitamin A signalling in sleep regulation

A further biological rhythm in which vitamin A plays a key role is the daily cycle of sleep. Humans spend approximately one-third of their lives sleeping, but the purpose of this vital process remains obscure. Sleep is characterized by measurable differences in brain activity of which slow-wave sleep (SWS, or non-rapid eye movement, NREM, sleep) featuring an electroencephalogram (EEG) delta oscillation of < 2 Hz is most associated with CNS maintenance and repair (Sei 2008). The power of the delta oscillation is a reliable predictor of the homeostatic requirement for sleep after an extended period of wakefulness, and is closely related to the recovery function of sleep (Maret *et al.* 2005). Indeed, Tononi and Cirelli (2006) have proposed a synaptic homeostasis hypothesis for the purpose of the delta wave in sleep. Briefly, wakefulness and daily activity are both linked to synaptic potentiation in cortical circuits owing to our interaction with the environment leading to increased cortical activation. This synaptic potentiation is associated with the homeostatic regulation of sleep in which increasing length of wakefulness greatly increases the power of the delta oscillation during sleep. When sleeping, we are not interacting with the environment around us and this results in a net downscaling of synaptic strength. Synaptic downscaling could have many benefits to brain function such as reducing the energy expenditure of neural circuits (Atwell and Laughlin 2001), reducing the space required for potentiated synapses (Eyre *et al.* 2003), and improving the memory function of cortical neurons during further wakeful periods (Huber *et al.* 2004). There is the intriguing possibility that this synaptic downscaling may be related to the role of RA in the process of homeostatic synaptic plasticity, which provides a homeostatic mechanism of neural circuit adaptation (Chen *et al.* 2012).

A definitive role for vitamin A signalling however is evident in the regulation of delta oscillations. This was first proposed by Maret (Maret *et al.* 2005), who observed that the relative contribution of the delta wave to SWS is determined by the RA receptor RARβ_1_. Moreover, vitamin A deficiency is known to significantly reduce the power of the delta oscillation in mice (Kitaoka *et al.* 2007), an effect that is reversed by sleep deprivation. Intriguingly, this vitamin A deficiency-induced reduction in delta power is accompanied by decreased dopamine metabolism in the striatum, an observation suggestive of a role for vitamin A in dopaminergic neurotransmission (Kitaoka *et al.* 2007). Indeed, the RA antagonist LE540 induced a significant reduction in wakefulness and delta oscillation power in mice treated over a period of 4 weeks. This was associated with a reduction in protein expression of the dopamine receptor D1DR in the striatum, and also tyrosine hydroxylase in the hippocampus (Kitaoka *et al.* 2011). It is possible therefore that retinoids may reduce wakefulness indirectly via RAR interaction with dopamine neurotransmission.

Further clues as to the involvement of vitamin A in the regulation of delta oscillations come from the study of sleep in mouse models of ageing. The SAMP8 senescence-accelerated prone mouse shows age-related deterioration in the sleep/wake cycle in comparison with the related senescence-accelerated resistant mouse (SAMR1; Butterfield and Poon 2005). EEG recordings from SAMP8 mice show a significantly reduced delta power in comparison with that of the SAMR1 mouse, accompanied by increased wakefulness, reduction in NREM sleep and reduction in cortical acetylcholine content (Kitaoka *et al.* 2010). The expression of several components of the retinoid cascade is reduced in the hippocampus and brainstem of the SAMP8 mouse including a highly significant reduction in the expression of RARα in the hippocampus, suggesting that a reduction in vitamin A signalling may be responsible in part for the decline in sleep observed in this model. Surprisingly, however, the RAR agonist AM80 does not improve delta oscillation power, and no improvement in rapid eye movement (REM) sleep is observed. It is likely therefore that the SAMP8 phenotype is only partially explained by deficiencies in the RAR activation (Kitaoka *et al.* 2010).

It is thus clear that vitamin A deficiency disrupts SWS through interruption of the delta oscillation in mice. However, given that specific RAR activation is unable to recover sleep abnormalities associated with age, it may be the case that vitamin A regulates sleep indirectly via interaction with dopamine and acetylcholine neurotransmission. Furthermore, vitamin A signalling and the molecular clock show significant cross-regulation in the hippocampus. Given the importance of this brain region to learning and memory, vitamin A may be an important factor in maintaining optimal molecular performance throughout the solar day.

## Conclusions

The observations described above make it clear that vitamin A status has an influence on the mammalian biological clock, impacting on the body's response to both the subjective day and night, and also on seasonal regulation of physiology. A key function of circannual rhythms is to anticipate favourable times of year for seasonal behaviours such as reproduction and hibernation, and nutrients are known to inform the clock about the quality and availability of food in the environment. It is clear that vitamin A is a key nutrient in this function.

## Abbreviations

ARAT: acyl-CoA:retinol acyltransferase
AVP: arginine vasopressin
BDNF: Brain-derived neurotrophic factor
BMAL: brain and muscle ARNT (arylhydrocarbon receptor nuclear translocator)-like protein
ChIP: chromatin immunoprecipitation
CLOCK: circadian locomotor output cycles kaput
CNS: central nervous system
CRABP: cellular retinoic acid-binding protein
CRBP: cellular retinol binding protein
CRY: cryptochrome
CYP: cytochrome P450
DHRS: dehydrogenase/reductase (SDR family)
DKK3: Dickkopf 3
DR: direct repeat
EEG: electroencephalogram
ERK: extracellular signal-regulated kinase
ES: embryonic stem
GABA: gamma-aminobutyric acid
GFAP: glial fibrillary acidic protein
GLUR1: glutamate receptor 1
LBD: ligand binding domain
LIF: leukaemia inhibitory factor
LRAT: lecithin:retinol acyltransferase
MAPK: mitogen-activated protein kinase
MOP: member of PAS protein
MSK1: mitogen- and stress-activated protein kinase-1
NADPH: nicotinamide adenine dinucleotide phosphate reduced
NREM: non-rapid eye movement
PER: period circadian clock
pSTAT: phosphorylated Signal transducer and activator of transcription 3
RA: retinoic acid
RALDH: retinaldehyde dehydrogenases
RAR: retinoic acid receptors
RARE: retinoic acid response element
RBP: retinol binding protein
RDHs: retinol dehydrogenases
REM: rapid eye movement
ROR: retinoid-related orphan receptor
RPE65: retinal pigment epithelium-specific protein 65kDa
RXR: retinoid X receptor
SCG: superior cervical ganglion
SCN: suprachaiasmatic nucleus
SFRP2: Secreted frizzled-related protein 2
STRA6: stimulated by retinoic acid 6
SWS: slow-wave sleep
TTR: transthyretin
VAD: vitamin A-deficient
